# Distinct fiber-specific white matter reductions pattern in early- and late-onset Alzheimer’s disease

**DOI:** 10.18632/aging.202702

**Published:** 2021-04-30

**Authors:** Xiao Luo, Shuyue Wang, Yeerfan Jiaerken, Kaicheng Li, Qingze Zeng, Ruiting Zhang, Chao Wang, Xiaopei Xu, Dan Wu, Peiyu Huang, Minming Zhang, Alzheimer’s Disease Neuroimaging Initiative (ADNI)

**Affiliations:** 1 Department of Radiology, The 2nd Affiliated Hospital of Zhejiang University School of Medicine, Hangzhou, China; 2 Key Laboratory for Biomedical Engineering of Ministry of Education, College of Biomedical Engineering and Instrument Science, Zhejiang University, Hangzhou, China

**Keywords:** fixel-based analysis, early-onset Alzheimer's disease, diffusion magnetic resonance imaging, white matter, amyloid deposition

## Abstract

Background: The underlying white matter impairment in patients with early and late-onset Alzheimer’s disease (EOAD and LOAD) is still unclear, and this might due to the complex AD pathology.

Methods: We included 31 EOAD, 45 LOAD, and 64 younger, 46 elder controls in our study to undergo MRI examinations. Fiber density (FD) and fiber bundle cross-section (FC) were measured using fixel-based analysis based on diffusion weighted images. On whole brain and tract-based level, we compared these parameters among different groups (p<0.05, FWE corrected). Moreover, we verified our results in another independent dataset using the same analyses.

Results: Compared to young healthy controls, EOAD had significantly lower FD in the splenium of corpus callosum, limbic tracts, cingulum bundles, and posterior thalamic radiation, and higher FC in the splenium of corpus callosum, dorsal cingulum and posterior thalamic radiation. On the other hand, LOAD had lower FD and FC as well. Importantly, a similar pattern was found in the independent validation dataset. Among all groups, both the FD and FC were associated with cognitive function. Furthermore, FD of fornix column and body, and FC of ventral cingulum were associated with composite amyloid and tau level (r=-0.34 and -0.53, p<0.001) respectively.

Conclusions: EOAD and LOAD were characterized by distinct white matter impairment patterns, which may be attributable to their different neuropathologies.

## INTRODUCTION

Alzheimer’s disease (AD) can be commonly categorized as either early onset (EOAD) or late onset (LOAD) based on an age cutoff of 65 years [1]. Comparing to LOAD, EOAD has relatively more aggressive disease course and shorter survival time [2], and their clinical symptoms are usually more occult despite occurring at a younger age. In addition to memory deficits, EOAD has lower performance in attention, visuospatial skills, and executive functions than LOAD [3, 4]. Although both EOAD and LOAD share the same neuropathological hallmarks (i.e., amyloid plaques and neurofibrillary tangles), distinct distribution patterns were found by previous post-mortem and *in vivo* imaging studies [5, 6]. Surprisingly, EOAD patients have a higher burden of amyloid deposition and neurofibrillary tangles than LOAD in frontal and parietal lobes [7–9], which is incompatible with their aging process.

Although AD is famous for cortical neurodegenerative pathology, recent studies using conventional diffusion tensor imaging (DTI) technique have also implicated white matter (WM) abnormalities in the risk and progression of AD [10–13]. Canu et al. also found that LOAD had altered fractional anisotropy (FA) and mean diffusivity (MD) in the posterior cingulum, corpus callosum, and temporal lobes, while EOAD had more diffuse WM abnormalities [14]. Although past studies have shed light on the investigation of WM degeneration in EOAD, the interpretation of diffusion imaging results was still greatly affected by the complexity of fiber bundle geometry considering crossing fibers account for up to 90% of whole brain WM voxels. Unfortunately, conventional diffusion tensor model cannot represent multiple, independent intra-voxel orientations thus fails to fit complex crossing fibers [15]. For instance, if a voxel contains multiple crossing fibers with different WM component, conventional DTI model can only describe the local FA, MD measures, each while microstructural (or macrostructural) information which is specific to the fiber orientation cannot be quantified. Since AD shows different forms of WM degeneration such as fiber atrophy (macroscopic level) and demyelination (microscopic level), the inability of DTI to capture different WM alterations complicates the interpretation of WM abnormalities thus further limits the use of this conventional method in neurodegenerative disease like AD. In short, the voxel-averaged metrics derived from DTI is neither fiber-specific nor easily interpretable.

To fill in the blanks, a novel diffusion model named fixel-based analysis (FBA) has been proposed in recent years [16]. The term “fixel” represents all the different fiber bundles with different orientations within a “voxel”. Each fixel carriers microstructural or macrostructural information, which is specific to the fiber orientation. The commonly investigated metrics of fixel-based analysis are fiber density (FD) and fiber cross-section (FC), reflecting the fibers density within a fiber bundle and macro-structural property of fiber bundles, respectively [17]. Additionally, fiber density and fiber cross-section (FDC) incorporates both the microscopic and macroscopic degenerative processes [18, 19]. A recent work showed that FBA could accurately reflect the microstructural differences between AD and healthy elderly, and more importantly, these results are biologically interpretable [18]. To date, this advanced method has been successfully applied in various neurodegenerative diseases, including AD and Parkinson disease [17, 18, 20].

In this cross-sectional study, FBA was applied to two independent datasets of EOAD and LOAD. We aimed to 1) investigate the white matter impairments in EOAD and LOAD; 2) establish the relation between FBA metrics like FD, FC, FDC, and other AD related measures including cognitive function and AD neuropathologies. Considering the distinct clinical and neuropathological features of EOAD and LOAD [6–9], we hypothesized that LOAD has a preferential loss in fiber bundles connecting to the medial temporal regions, while EOAD has more widespread impairments in WM tracts.

## RESULTS

### Demographics

AD patients and control subjects did not differ significantly in age, sex and education. For each dataset, there is no significant difference between EOAD and LOAD, or YHC and OHC in general cognitive score (p > 0.05). Furthermore, there is no interaction relationship between the onset age (<65 or ≥ 65 years) and the disease status (whether healthy or not) (p > 0.05). Notably, patients from the ADNI dataset had milder disease severity and higher education level than those patients from the ZJU dataset (Supplementary Material 1).

### Whole-brain fixel-based analysis results

### ZJU database

**Figure 1:**
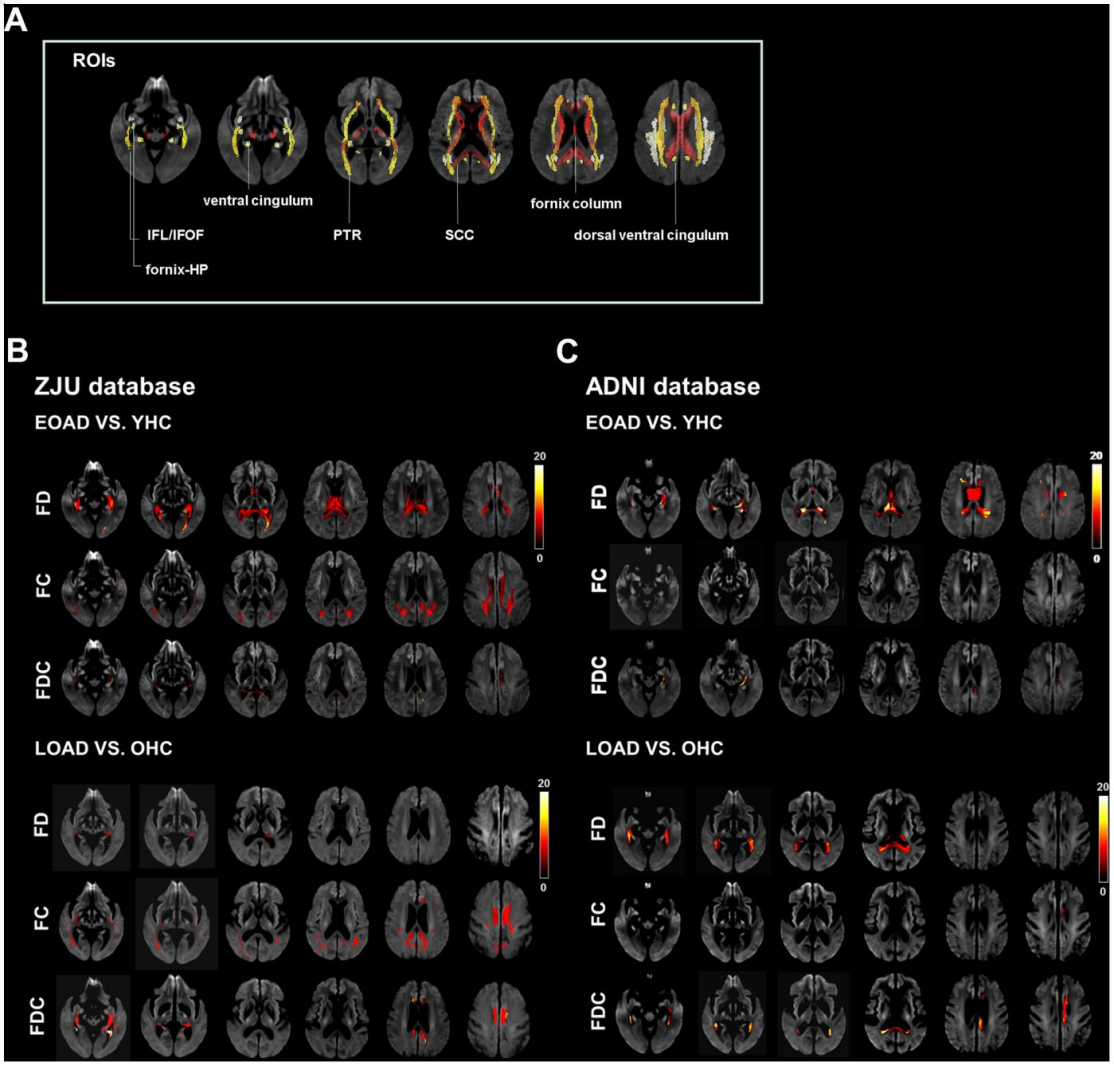
**Illustrates the location reference and fiber tract-specific reduction in EOAD/LOAD versus controls from whole-brain fixel-based analysis.** (**A**) illustrates the location reference. (**B**) and (**C**) Represent results from ZJU and ADNI databases, respectively. We color-coded the significant streamlines by the effect size expressed as a percentage relative to the control groups. Abbreviations: ZJU, Zhejiang University; FD, fiber density; FC, fiber bundle cross-section; FDC, fiber density and bundle cross-section.

EOAD had a diffuse decrease of FD in SCC, column and body of fornix, left fornix-HP, bilateral dorsal/ventral cingulum, and PTR; additionally, EOAD had a decrease of FC in SCC, bilateral dorsal cingulum, and PTR. Regarding the composite index (FDC), EOAD had a decrease of FDC in the SCC, left fornix-hippocampus, bilateral dorsal/ventral cingulum, and PTR as compared to YHC. We also found that LOAD had a decrease of FD in the bilateral dorsal/ventral cingulum and left ILF/IFOF and a decrease of FC in the SCC, bilateral dorsal/ventral cingulum, PTR, and ILF/IFOF as compared to OHC. Moreover, LOAD had a decreased of FDC in the SCC, bilateral dorsal/ventral cingulum, ILF/IFOF, and PTR (Figure 1).

### ADNI database

EOAD had significantly lower FD in the CC, column and body of fornix, and left ventral cingulum as compared to YHC, while there is no significant difference in FC was found between EOAD and YHC.

EOAD had lower FDC in the left ventral cingulum. On the other hand, LOAD had lower FD in the SCC, column and body of fornix, right fornix-hippocampus, and left ventral cingulum as compared to OHC. In addition, LOAD had lower FC in the column and body of fornix, left dorsal cingulum, and lower FDC in the SCC and left dorsal/ventral cingulum (Figure 1).

### Repeatability test of whole-brain fixel-based analysis results

To eliminate the potential statistical bias due to differences in patient demographics from two independent datasets, we matched two datasets based on their age, sex and basic demographics. After conducting the same analysis on this combined dataset, the results we yielded were with similar patterns despite of the fact that the altered areas we detected were smaller in combined dataset compared to previous mentioned results (Supplementary Material 2).

Besides, AD is a multifactorial disease with multiple contributors to its pathophysiology, including cerebral small vascular disease (CSVD) [21]. Recent work suggested that the effects of CSVD on WM integrity should also be accounted for [22]. After adjusting for WMH, we repeated the same analyses in both ZJU and ADNI datasets and the results we yielded remained mostly unchanged despite of a lower but statistically sound significance level (Supplementary Material 3). In general, our results were consistent with recent findings and suggested that to some extent, WMH did contribute to part of the microstructural alterations in AD patients.

### Tract level analysis

### ZJU database

**Figure 2:**
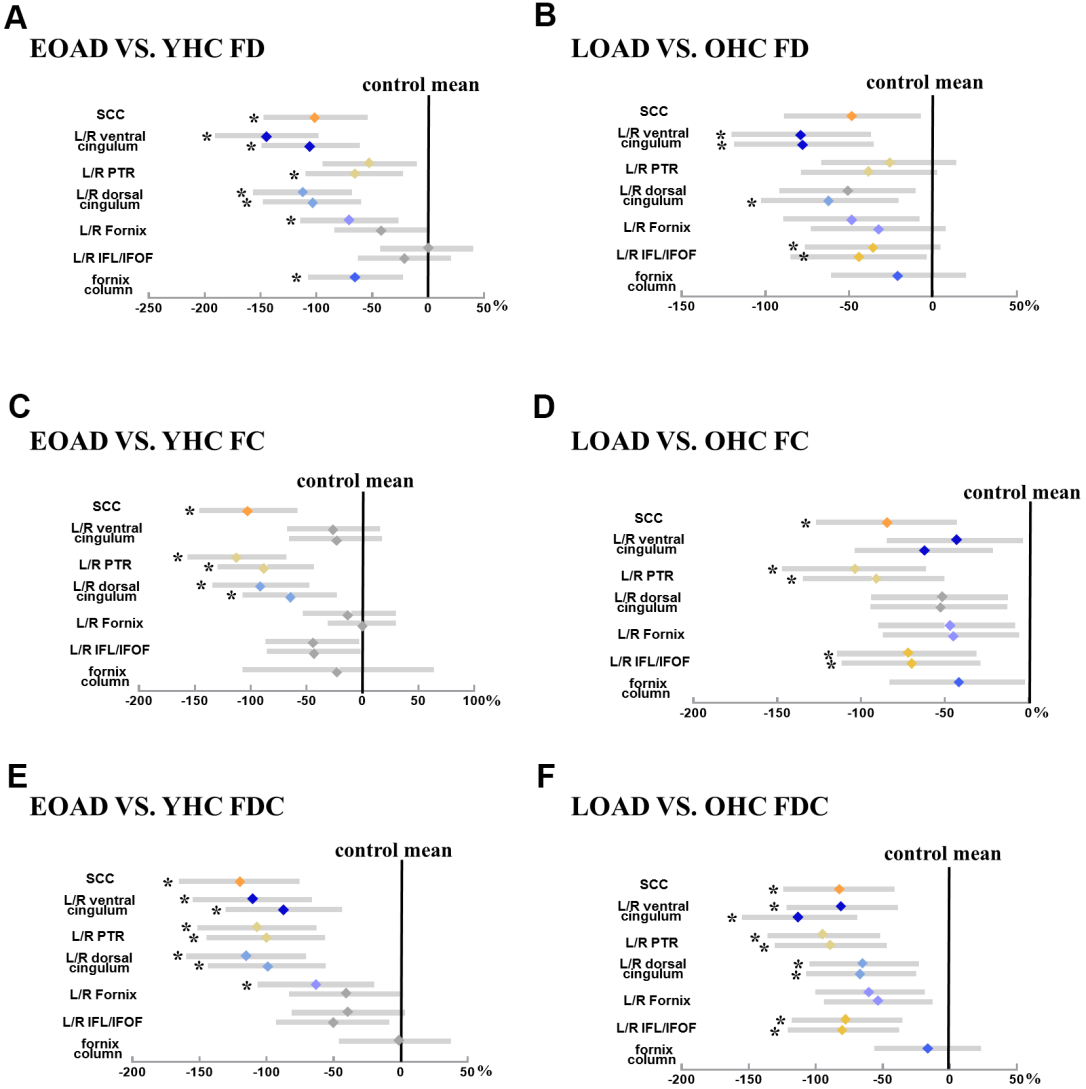
**Illustrates the group difference (patient VS. control) in mean FD, FC, and FDC based on the ZJU database.** (**A**, **C**, **E**) Represents the mean FD, FC, and FDC (diamond) and 95% CI (bars) within tracts of interest are displayed for early-onset Alzheimer's disease groups, respectively; (**B**, **D**, **F**) represents the mean FD, FC, and FDC (diamond) and 95% CI (bars) within tracts of interest are displayed for late-onset Alzheimer's disease groups, respectively. The more the color bar shifted to the left, representing more significant difference from healthy controls. Notably, significant tracts (Bonferroni-corrected P-value < 0.05, controlling for age and sex) are marked with star symbols. Abbreviation: SCC, splenium of the corpus callosum; ILF/IFOF, inferior longitudinal fasciculus/inferior frontal-occipital fasciculus; PTR, posterior thalamic radiation; HP, hippocampus; FD, fiber density; FC, fiber bundle cross-section; FDC, fiber density and cross-section.

EOAD had lower mean FD in the SCC, fornix column and body, bilateral dorsal/ventral cingulum, right PTR, left fornix-hippocampus, as well as lower mean FC in the SCC, bilateral dorsal cingulum and PTR compared to YHC. Moreover, EOAD had lower mean FDC in the SCC, bilateral cingulum bundles and PTR, and left fornix-hippocampus compared to YHC. On the other hand, LOAD had lower mean FD in the right dorsal cingulum, bilateral ventral cingulum, ILF/IFOF, as well as lower mean FC in the SCC, bilateral ILF/IFOF and PTR compared to OHC. In addition, LOAD demonstrated decreased mean FDC in the SCC, bilateral cingulum bundles, ILF/IFOF, and PTR when compared to OHC (Figure 2).

### ADNI database

EOAD had lower mean FD in the column and body of fornix and left ventral cingulum. However, there is no difference in mean FC and FDC between EOAD and YHC. On the other hand, comparing to YHC, LOAD had significantly decreased mean FD in the column and body of fornix, bilateral ventral cingulum, as well as decreased mean FC in the column and body of fornix. LOAD also had decreased FDC in left cingulum HP.

### Relationship between fixel-based metrics and cognition/neuropathologies

**Figure 3:**
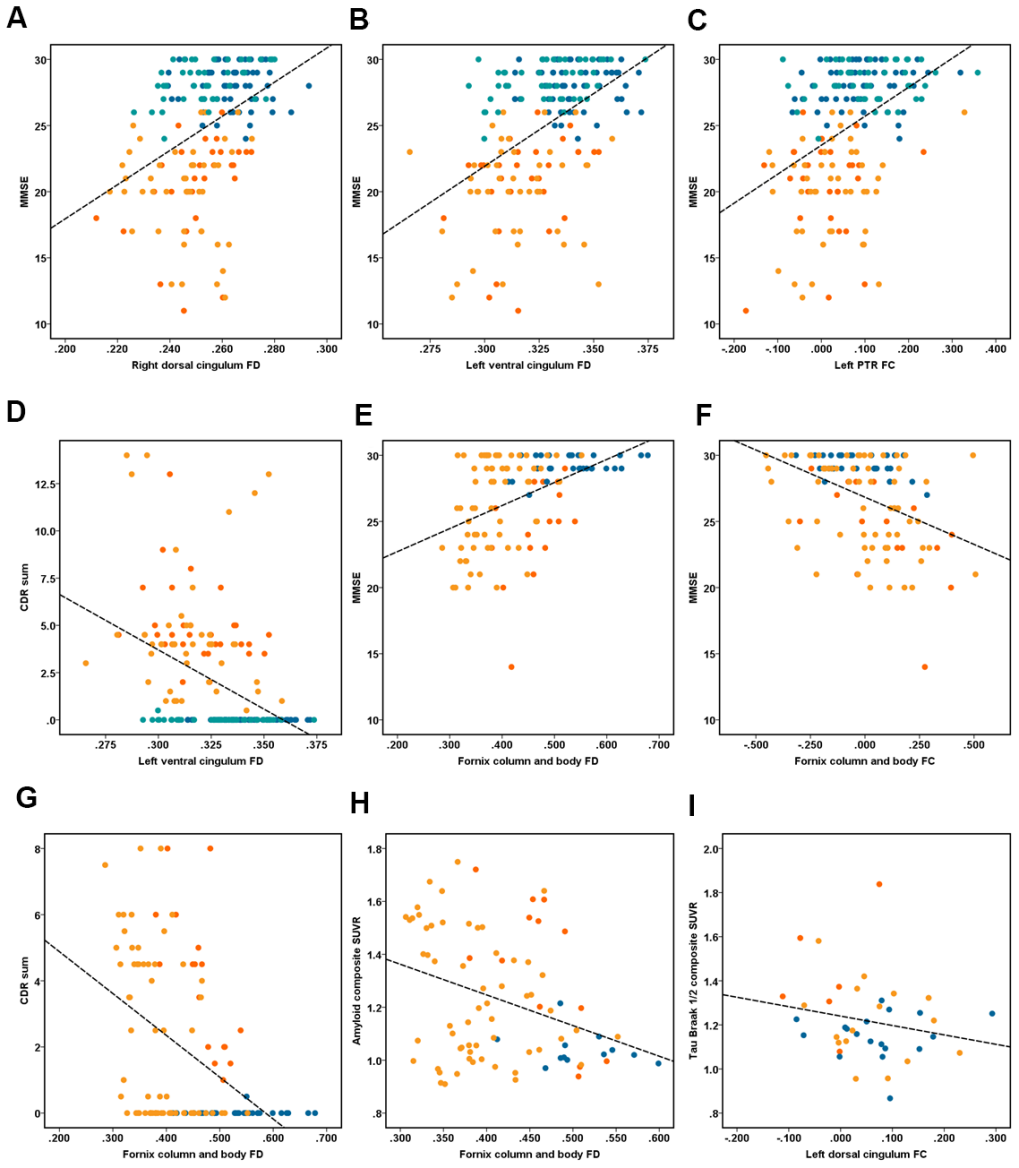
**Illustrates the association between fixel-based analysis metrics and clinical data.** Correlation analyses of (**A**–**D**) performed in the ZJU database. (**A**) Right dorsal cingulum fiber density (FD) related with MMSE (r = 0.40, P < 0.001); (**B**) left ventral cingulum FD related with MMSE (r = 0.48, P < 0.001); (**C**) left PTR fiber bundle cross-section (FC) related with MMSE (r = 0.43, P < 0.001); (**D**) left ventral cingulum FD related with CDR sum (r = 0.42, P < 0.001). Correlation analyses of (**E**–**I**) performed in the ADNI database. (**E**) Fornix column and body FD related with MMSE (r = 0.45, P < 0.001); (**F**) fornix column and body FC related with MMSE (r = -0.42, P < 0.001); (**G**) fornix column and body FD related with CDR sum (r = -0.45, P < 0.001); (**H**) fornix column and body FD related with composite amyloid SUVR (r = -0.34, P < 0.001); (**I**) right ventral cingulum FC related with tau Braak I/II composite SUVR (r = -0.53, P < 0.001). Note: dot of red, orange, dark blue and light blue represent the early-onset Alzheimer’s disease (EOAD), late-onset Alzheimer’s disease (LOAD), young healthy controls (YHC), and old healthy controls (OHC).

Among all groups (EOAD, YHC, LOAD, and OHC), we correlated both the mean FD and FC with the cognitive assessment (Figure 3). Here we only displayed the significant associations between FBA metrics (i.e., FD and FC) and total MMSE/CDR. With the ADNI dataset, we also correlated the FBA metrics with PET-derived AD neuropathological markers. Correlation results in subgroup level were shown in Supplementary Material 4.

### ZJU database

We found that the MMSE was associated with the FD in the SCC (r = 0.33, P < 0.001), bilateral dorsal cingulum (r = 0.40/0.38, P < 0.001), left ventral cingulum (r = 0.48, P < 0.001), bilateral ILF/IFOF (r = 0.23/0.25, P < 0.001), while the total CDR was associated with the FD in the SCC (r = -0.31, P < 0.001), bilateral dorsal cingulum (r = -0.28/-0.26, P < 0.001), left ventral cingulum (r = -0.42, P < 0.001). On the other hand, we found that the MMSE was correlated with the FC in the SCC (r = -0.35, P < 0.001), bilateral dorsal cingulum (r = 0.26/0.33, respectively, P < 0.001), ILF/IFOF (r = 0.28/0.25, P < 0.001), and PTR (r = 0.39/0.43, respectively, P < 0.001), while the total CDR was correlated with the FC in the SCC (r = -0.29, P < 0.001), left dorsal cingulum (r = -0.28, P < 0.001), and bilateral PTR (r = -0.32/-0.35, P < 0.001, Figure 3).

### ADNI database

We found that the MMSE was correlated with the FD in the SCC (r = 0.33, P < 0.001), bilateral dorsal cingulum (r = 0.40/0.38, respectively, P < 0.001), left ventral cingulum (r = 0.48), and bilateral ILF/IFOF (r = 0.23/0.25, P < 0.001), while the total CDR was correlated with the FD in SCC (r = -0.31, P < 0.001), bilateral dorsal cingulum (r = -0.28/-0.26, P < 0.001), and left ventral cingulum (r = 0.42, P < 0.001). On the other hand, the MMSE was correlated with the FC in the SCC (r = 0.35, P < 0.001), bilateral dorsal cingulum (r = 0.26/0.33, P < 0.001), ILF/IFOF (r = 0.28/0.25, P < 0.001), PTR (r = 0.39/0.43, P < 0.001); while the total CDR was correlated with the FC in the SCC (r = -0.29, P < 0.001), left dorsal cingulum (r = -0.28, P < 0.001), and bilateral PTR (r = -0.32/-0.35, respectively, P < 0.001, Figure 3).

### Associations between FBA metrics and PET data

**Figure 4:**
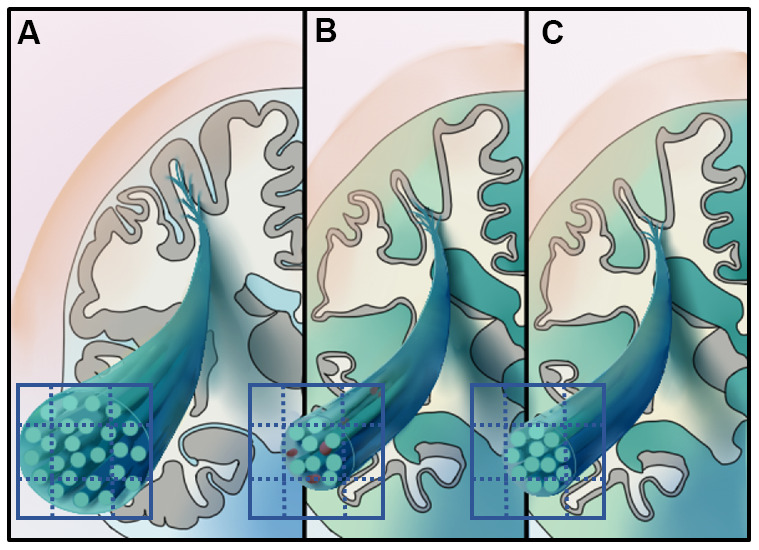
**Illustrates the hypothetical model of white matter pathological processes in early-onset Alzheimer’s disease (EOAD) and late-onset Alzheimer's disease (LOAD).** Specifically, 3D schematic represents a magnified fiber bundle, green tubule and grid on the cross-section represent the axons and imaging voxels, respectively. (**A**) Represents the healthy fiber bundle in the aging population. EOAD (**B**) might undergo white mater disruption involved both the microstructural and macrostructural level, while the macrostructural degeneration dominates white matter loss in LOAD (**C**).

Among all groups, the FD of column and body of fornix was correlated with the composite amyloid deposition SUVR (r = -0.34), while the FC of right ventral cingulum was correlated with the mean tau retention of Braak I-II ROI, including the bilateral entorhinal and hippocampus (r = -0.53). Figure 4 illustrates the hypothetical model of white matter pathological processes in EOAD and LOAD.

## DISCUSSION

In current study, we used FBA to study the WM impairments in EOAD and LOAD and further validated our results on another entirely independent dataset. The major findings include: (i) LOAD had severer but more spatially confined WM impairments along the limbic-related tracts, while EOAD had more widely distributed WM impairments involving the limbic-related tracts, the column and body of fornix, left fornix-HP, SCC, and PTR even after correcting for WMH burden; (ii) the WM impairment we found in EOAD was majorly characterized by decreased FD rather than FC in both datasets; (iii) both FD and FC were associated with the cognitive function and greater disease burden. More importantly, based on the ADNI database, the decreased FD and FC were associated with increased SUVR of amyloid and tau, respectively. Conclusively, our results suggested that EOAD and LOAD were featured by distinct WM impairment patterns thus may had different pathological mechanisms.

### Distinct white matter impairment pattern in LOAD and EOAD patients

White matter impairments in LOAD were mostly located on the bilateral dorsal and ventral cingulum and ILF/IFOF in both datasets. Notably, white matter impairments of LOAD were more pronounced, though more spatially confined than EOAD. This result is consistent with past evidence that AD patients with a younger onset age have less pathology in the hippocampus but more extensive involvement of cortex [23]. Anatomically, the cingulum bundle connects the anterior medial prefrontal cortex, PCC, and medial temporal lobe, which are the hubs of default mode network (DMN) [24]. Besides, bilateral ILF/IFOF directly links the angular gyrus with the DMN [25]. Our results thus suggested that white matter tracts subserving the DMN are especially vulnerable to the effects of AD pathologies. Similarly, previous studies reported that LOAD patients were featured by restricted DMN disconnection [26–28].

On the contrary, EOAD patients exhibited additional white matter impairments in the column and body of fornix, left fornix-hippocampus, SCC, and PTR. Previous work also showed that EOAD patients have more widespread white matter microstructural impairment than LOAD [14, 29]. Our results are also supported by previous functional imaging evidence which showed that EOAD has various network dysfunction involving executive control, visuospatial, language, and memory-related networks [26–28]. Anatomically, the crura of fornix lies along the concavity of the hippocampus while the remainder is continued as the fimbria of hippocampus, which is prolonged into the uncus of the parahippocampal gyrus. SCC comprises the homotopic connections between the bilateral parietal and posterior cingulate cortices, which further form the executive-related network [30]. PTR connects the thalamus with the visual cortex, acting as the anatomical foundation of visuospatial and language function [31]. As we found the correlation between fixel-based metrics and multi-domain cognitive functions, we thus speculated that the widespread white matter impairment we found accounts for the atypical symptoms in EOAD. Notably, after adjusting for WMH, our results show that the FBA results remained mostly unchanged in both ZJU and ADNI datasets, but the significance level somehow lowered. Our results suggested that WMH did contribute to part of the microstructural alterations in AD patients, which is in accordance with previous results [22]. Thus, it is necessary to take CSVD burden into account in future AD studies.

### White matter damage of EOAD is featured by reduced FD

In the ZJU dataset, EOAD showed a decrease of both FD and FC, while in LOAD, the decrease of FC was more salient than FD. A decrease in FD usually represents lower volume fraction within a voxel, while a decrease in FC represents smaller cross-sectional area of fiber bundle's [16]. Thus, decrease in both FD and FC may represent two pathological processes. Reasonably, the white matter disruption in EOAD may result from the deposition of amyloid plaques and neurofibrillary tangles. In contrast, the white matter impairments in LOAD may largely result from neurofibrillary tangles. This hypothesis is supported by our correlation analysis which showed that FD and FC were associated with amyloid deposition and tau retention respectively. Our speculations are in line with previous neuropathological findings that EOAD had a higher burden of AD neuropathologies than LOAD. Additionally, in both EOAD and LOAD, we found that white matter tracts with lower FD (i.e., fornix-related tracts) were anatomically connected with the DMN, which is the region first affected by amyloid deposition pathologies. In contrast, tracts with lower FC are long projection fibers (e.g., PTR) from posterior cortices. This is consistent with past evidence that parietal white matter impairment in AD was caused more by neurofibrillary tangles than amyloid plaques [32].

### Discrepancies between results from different datasets

Although most results we got from different datasets showed the same trend, there are still discrepancies even after matching the sample. In the ADNI dataset, the extent of FD and FC decrease is smaller than that from the ZJU dataset. Additionally, LOAD in ADNI demonstrated salient decrease in FD rather than FC, and vice versa for ZJU. Several reasons may contribute for this: first, the clinical symptoms for patients from the ADNI dataset are milder than that of the ZJU dataset. Accordingly, white matter tracts involved in the early AD continuum, such as cingulum bundles and fornix-related tracts, had impairments of similar extent across datasets [33]. Second, brain reserve may delay the neuropathological process [34]. Moreover, patients from ADNI dataset had a higher education level than that of the ZJU dataset.

There are also several limitations of our study. First, higher number of diffusion gradient directions and higher b-values might help to estimate intra-axonal volume fraction and multiple fiber orientations. Although the low b-value should not hold much influence over the FC, and FBA have previously been applied in neurological disorders using diffusion data with lower b-value [35–38] and lower gradient directions as well [17, 20]. Based on the results from test-retest analysis, we believe our study showed the possibility of applying advanced diffusion-based techniques to data acquired under clinical settings, which help probe different neurodegenerative processes. Second, FBA assumed that fiber orientation distribution function response is the same for all fiber populations. However, it is not only the intra-axonal space that will contribute to the diffusion signal, but the diffusivity or tortuosity of the extracellular space will also be confounded to the FOD amplitudes, which needs to be further validated. Third, based on the 2018 AD research framework, ATN biological diagnosis criteria is recommended to diagnose AD patients [39].

Conclusively, we investigated the white matter impairment pattern in EOAD and LOAD. We found that EOAD had more widespread white matter microstructural impairment than LOAD, which may contribute to their worse cognitive profiles. White matter microstructural and macrostructural impairments were respectively associated with amyloid and neurofibrillary tangles, implicating that the white matter of EOAD are more susceptible to amyloid deposition.

## MATERIALS AND METHODS

### Participant and neuropsychological assessment

Each participant underwent MRI scanning and neuropsychological evolutions, parts of them additionally underwent amyloid/tau PET scanning. In the database of Zhejiang University (ZJU), the diagnosis of probable AD was made by an experienced neurologist according to the NINCDS/ADRDA criteria. Additionally, the neurologist evaluated the neurological history, blood biochemical examination, and neuropsychological scales to exclude dementia from other causes. The age at disease onset was provided by the patient’s family members or caregivers during interview. Regarding the Alzheimer’s Disease Neuroimaging Initiative (ADNI) dataset, neurologists from multiple cooperation institutes made the AD diagnosis. Consistent with previous studies, we dichotomized AD patients into early- and late-onset groups (age at onset <65 or ≥ 65 years, respectively) [8, 14]. All participants underwent the evaluations of Mini-Mental State Examination (MMSE) and neuropsychological battery, involving Wechsler Memory Scale-logical memory (WMS-LM), delayed recall, language (Semantic verbal fluency, SVF), attention (Trail Making Test, Part A, TMT-A), and executive function (Trail Making Test, Part B, TMT-B). Additionally, dementia severity and depression severity were assessed based on Clinical Dementia Rating (CDR) and the Geriatric Depression Scale (GDS).

We divided healthy elderly controls into younger and older group based on their age (YHC/OHC, age <65 or ≥ 65 years, respectively). Notably, age, education, and sex of YHC and OHC were matched to their corresponding patient group respectively. In both datasets, we defined YHC and OHC as having a CDR of 0, an MMSE between 24 and 30 (inclusive), a WMS-LM, delayed recall (≥ 9 for subjects having ≥ 16 years education; ≥ 5 for subjects having 8–15 years education; and ≥ 3 for subjects having ≤ 7 years education); absence of clinical depression (GDS < 6) and dementia symptom. We excluded subjects with following conditions: significant neurological, psychiatric, and medical illness; severe head trauma history [40]; taking non-AD-related medication that may potentially influence cerebral function; clinical depression; drug or alcohol abuse.

Finally, we identified 31 EOAD, 45 LOAD, 64 YHC, and 46 OHC from the ZJU dataset (Table 1), as well as 17 EOAD, 30 LOAD, 31 YHC, and 34 OHC from the ADNI dataset (Supplementary Material 1).

**Table 1:** Clinical and demographic data of the ZJU database.

**Group**	**YHC**	**EOAD**	**OHC**	**LOAD**	**Interaction**		**ANOVA**	
	**n=64**	**n=31**	**n=46**	**n=45**	**F/χ^2^**	**p**	**F/χ^2^**	**p**
Age	59.7 (2.5)	60.8 (3.3)	72.4 (3.8)	74.3 (4.6)	0.7	0.4	86.9	**<0.001**
Education	10.7 (3.7)	9.4 (2.9)	10.5 (4.1)	10.5 (4.0)	2.6	0.1	0.9	0.4
Sex, F/M	38/26	21/10	22/24	23/22	0.1	0.8	3.7	0.3
GDS	1.9 (2.5)	1.6 (1.5)	1.2 (1.6)	1.4 (1.2)	0.7	0.4	1.2	0.3
CDR global	0 (0)	1.1 (0.3)ƚ	0 (0)	1.0 (0.5)ǂ	0.2	0.6	220.9	**<0.001**
CDR sum	0 (0)	4.9 (2.1)ƚ	0 (0.1)	4.7 (3.6)ǂ	0.1	0.7	87	**<0.001**
MMSE	28.1 (1.6)	20.6 (3.6)ƚ	28.3 (1.5)	20.0 (3.7)ǂ	0.7	0.4	140	**<0.001**
LM delay	8.5 (4.3)	0.6 (1.3)ƚ	8.6 (3.7)	0.3 (0.7)ǂ	0.01	0.9	96	**<0.001**
TMT-A	69.2 (28.2)	98.3 (40.6)ƚ	70.8 (29.8)	106.3 (38.0)ǂ	0.4	0.6	16.5	**<0.001**
TMT-B	172.6 (64.3)	223.6 (90.6)ƚ	181.5 (69.8)	250.3 (83.4)ǂ	0.1	0.7	17.8	**<0.001**
SVF	16.3 (3.9)	11.0 (5.3)ƚ	16.0 (4.8)	8.9 (5.0)ǂ	3.6	0.1	31.3	**<0.001**
CDT	4.1 (0.7)	3.2 (0.6)ƚ	4.3 (0.6)	3.3 (0.6)ǂ	0.2	0.7	33.6	**<0.001**

ƚ and ǂ Represent the significant difference between YHC and OHC, as well as EOAD and LOAD (p<0.05), respectively. Interactive effects comprise the factors of onset age (<65 or ≥65 years) and disease status (controls or patients). Abbreviations: YHC, young healthy controls; EOAD, early-onset Alzheimer's disease; OHC, old healthy controls; LOAD, late-onset Alzheimer's disease; CDR global/sum, Clinical Dementia Rating, global score and sum score of box; MMSE, Mini-Mental State Examination; GDS, Geriatric Depression Scale; LM, Logical Memory; TMT-A/B, Trail Making Test, part A/B; SVF, Semantic Verbal Fluency.

### MR imaging acquisition

In both databases, researchers used foam padding and earplugs to limit head motion and reduce scanner noise. Regarding the ZJU database, MRI data were acquired from the 3.0 Tesla scanner (GE Discover 750) using an 8-channel head coil. We acquired the T1-weighted structural images based on the fast-spoiled gradient-echo sequence with TR = 7.3 ms, TE = 3.0 ms; slice number = 196; FOV=256 ×256; voxel size=1.02 ×mm ×1.02 ×mm 1.2×mm; flip angle = 11°; and bandwidth = 244.141 Hz/pix. DWI data were acquired using a single shot, diffusion-weighted spin-echo echo-planar imaging sequence. Specifically, images were acquired using b = 1,000 s/mm^2^ in 30 directions; 5 volumes were acquired without diffusion weighting (b-value = 0 s/mm^2^). Other parameters of DTI were as follows: TR/TE = 8,000/80.8 ms, flip angle = 90°, slice thickness = 2 mm without slice gap, matrix size = 128 × 128, FOV = 256 × 256.

Regarding ADNI database, each subject underwent MRI scanning based on the ADNI protocol using the GE 3.0 Tesla scanner across 14 institute sites. The sequence of T1-weighted structural imaging is IR-SPGR sequence with following parameters: repetition time (TR) = 6.96 ms, echo time (TE) = 2.8 ms, Invert time (TI) = 400 ms, field of view (FOV) = 256 × 256, voxel size=1.02 mm × 1.02 mm × 1.2mm, flip angle = 11°. Diffusion-weighted imaging (DWI) data were acquired using the Echo Planar Imaging (EPI) sequence with 41 directions and the following specifications: slice number = 59, acquisition matrix = 256 × 256, voxel size= 1.4 mm × 1.4 mm × 2.7 mm, flip angle= 90°, with 41 diffusion-weighted images (b = 1000 s/mm^2^) and 5 non-diffusion weighted images (b = 0 s/mm^2^). Across 14 sites, the TR was the range from 12500 to 13000 ms.

### DWI pre-processing and Fixel-based analysis

The DWI data pre-processing and fixel-based analysis were performed using MRtrix3 (https://www.mrtrix.org) [41]. The DWI were denoised then corrected for eddy-current, head motion, and bias field; then, we normalized intensity across subjects. An average response function was firstly generated by averaging all subjects’ single fiber response function before the FBA. Then, fiber orientation distributions were estimated using constrained spherical deconvolution, and we applied multi-shell multi-tissue constrained spherical deconvolution to obtain more robust outcomes [42]. From between-group comparisons, we generated fiber orientation distributions template of young subjects based on randomly selected 10 EOAD and 10 YHC. Similarly, we generated the template for each elder subject. Then the fiber orientation distributions in the template were segmented to fixels for generating the fixel template analysis mask. For spatial correspondence, the fiber orientation distributions image of each subject was transformed into the corresponding template. Then we used the resulting local transformations to segment and reorient fixels to match the template in each voxel. Finally, we assigned FD, FC, and FDC to fixels in the template space.

To facilitate connectivity-based fixel enhancement, whole-brain probabilistic tractography was performed on a group-wised template of fiber orientation distributions. A total of 20 million streamlines was generated and filtered to 2 million for reducing reconstruction biases using the spherical deconvolution informed filtering of tractograms algorithm [43].

### PET image data

Based on predefined regions of interest (ROI), standardized update value ratios (SUVRs) were calculated using the mean signal of the whole cerebellar (florbetapir) and cerebellar cortex (flortaucipir) as the reference region [44]. We chose 4 ROI, including the SUVRs of composite amyloid deposition and Tau from Braak stage I/II, III/IV, and V/VI (Supplementary Material 5) [45].

### White matter hyperintensities assessment

Recently, increasing evidence shows that AD is a multifactorial disease with multiple contributors, including cerebrovascular disease [21]. Considering that WMH is the typical imaging marker of CSVD [46], we measured the WMH burden of each subject using a semi-quantitative scoring method developed by Fazekas et al. [47].

### Statistical analysis

### Analysis of demographics

We performed multiple statistical analyses on demographic data using SPSS (Version 23). Continuous variables were compared between groups using two-sample T-tests. Group differences in categorical variables were assessed using Fisher’s exact test.

### Fixel-based analysis

The FD, FC, and FDC of each subject in the fixel level (FWE corrected, P-values < 0.05, 5000 permutations, controlling age and sex) were compared between groups. We then performed connectivity-based smoothing and statistical inference using CFE (smoothing = 10 mm full-width at half-maximum, C = 0.5, E = 2, H = 3) [48]. Significant streamlines were color-coded by the effect size (percentage) relative to their corresponding controls.

### Tract-based analysis and correlation analysis

Based on previous literature, we focused on 12 tracts which are susceptible to AD-related pathologies, including the splenium of corpus callosum (SCC), fornix column and body, bilateral dorsal and ventral cingulum, inferior longitudinal fasciculus/inferior frontal-occipital fasciculus (ILF/IFOF), posterior thalamic radiation (PTR), and fornix-hippocampus [14, 18, 49, 50]. Then, we extracted the mean FD, FC, and FDC values from these pre-defined tracts and used two-sample t-tests to assess the differences of fixel-based analysis metrics between AD patients and counterpart, controlling for age and sex (Bonferroni-corrected, P-value < 0.05).

Furthermore, we explored the correlation between mean fixel-based analysis metrics and cognitive performances, as well as PET biomarkers. Given the explorative nature of our work, statistical significance is defined as p-value < 0.001 (uncorrected) controlling for age and sex.

### Ethics approval and patient consent statement

Regarding the ZJU database, our study was approved by the Review Board of the Second Affiliated Hospital, Zhejiang University School of Medicine, and conducted following the Declaration of Helsinki. We obtained the written informed consent from all participants. Regarding the ADNI database, this project was approved by the Institutional Review Boards of all participating institutions, and all of the participating institutions, and informed written consent was obtained from all participants at each site. More information could be found in http://adni.loni.usc.edu/.

## Supplementary Material

Supplementary Materials
