# Supplementary material for: Distinct fiber-specific white matter reductions pattern in early- and late-onset Alzheimer’s disease
Source: Aging (Albany NY). 2021 Apr 30;13(9):12410–30. doi: 10.18632/aging.202702 (PMC8148465; doi:10.18632/aging.202702)
Supplement: Supplementary Materials [file aging-13-202702-s001.pdf]

SUPPLEMENTARY MATERIALS

Supplementary Materials 1

Participants' information

Introduction of database of Zhejiang University and Alzheimer's Disease Neuroimaging Initiative

Regarding the Zhejiang University (ZJU) database, we recruited participants from the Second Affiliated Hospital of Zhejiang University School of Medicine, Zhejiang Province, P.R.China. This database was established in 2012. Alzheimer's disease (AD) and mild cognitive impairment patients were recruited from the memory clinics by neurologists, and healthy controls were recruited from the spouses of patients or community advertisements. Participants from the ZJU databased are entirely composed of the Chinese Han population, while participants from the ADNI database are mainly composed of the Caucasian population.

Each participant from the database of ZJU and ADNI underwent a comprehensive cognitive scale, blood collection, and multiple sequence MRI scanning [1]. Further, in the ADNI database, 13 out of 17 EOAD (76.5%), 27 out of 30 LOAD (90.0%), 12 out of 31 YHC (38.7%), and 32 out of 34 OHC (94.1%) had florbetapir PET data; 6 out of 17 EOAD (35.3%), 18 out of 31 YHC (58.1%), and 16 out of 34 OHC (47.0%) had flortaucipir-PET data. We aim to explore the neurobiological mechanisms of EOAD and find early AD imaging biomarkers.

Demographics

All participants underwent the evaluations of Mini-Mental State Examination (MMSE) [2] and neuropsychological battery, involving Wechsler Memory Scale-logical memory (WMS-LM), delayed recall, language (Semantic verbal fluency, SVF), attention (Trail Making Test, Part A, TMT-A), and executive function (Trail Making Test, Part B, TMT-B). Additionally, dementia severity and depression severity were assessed based on Clinical Dementia Rating (CDR) [3] and the Geriatric Depression Scale (GDS) [4]. In both databases, early-onset Alzheimer's disease (EOAD) and young healthy controls (YHC) matched late-onset Alzheimer's disease (LOAD) and old healthy controls (OHC), respectively, for the age, gender, education, general cognitive ability (reflected by MMSE), and disease severity (reflected by Clinical Dementia Rating, CDR). Notably, the interval between the behavioral scale and the MRI scan should not exceed one week for the ZJU database, and three months for the ADNI database.

In the ZJU database, the diagnosis of probable AD was made by an experienced neurologist according to the

Supplementary Table 1. Demographics of the groups in the ADNI database.

Group	YHC	EOAD	OHC	LOAD	Interaction		ANOVA	
	n=31	n=17	n=34	n=30	F/χ^2	p	F/χ^2	p
Age	61.9 (2.4)	61.2 (2.6)	74.4 (4.5)	76.8 (5.3)	0.7	0.4	107.6	**<0.001**
Education	16.6 (2.4)	15.5 (2.9)	16.2 (3.7)	15.4 (3.0)	0.5	0.5	0.9	0.4
Sex, F/M	23/8	11/6	22/12	11/19	0.04	0.8	9.9	**0.02***
GDS	0.6 (0.7)	2.4 (1.1)ł	0.9 (1.3)	1.9 (1.6)ǂ	0.6	0.4	11.9	**<0.001**
CDR global	0 (0)	0.8 (0.2)ł	0 (0)	0.9 (0.2)ǂ	0.1	0.8	272.6	**<0.001**
CDR sum	0 (0.1)	3.9 (2.2)ł	0.1 (0.2)	4.6 (1.6)ǂ	1.1	0.3	120.4	**<0.001**
MMSE	29.2 (0.8)	24.6 (3.8)ł	29.2 (1.0)	23.1 (1.9)ǂ	1.6	0.2	78.6	**<0.001**
LM delay	13.7 (3.9)	5.4 (3.7)ł	12.8 (3.0)	1.2 (1.6)ǂ	3.9	0.1	82.6	**<0.001**
TMT-A	28.7 (6.1)	64.4 (42.3)ł	35.9 (8.4)	53.3 (26.7)ǂ	0.04	0.9	9.6	**<0.001**
TMT-B	62.6 (17.1)	170.5 (99.6)ł	84.5 (34.7)	206.9 (95.7)ǂ	1.3	0.3	24.5	**<0.001**
SVF	24.4 (4.3)	16.4 (6.3)ł	20.5 (5.0)	12.4 (4.9)ǂ	0.005	0.9	25	**<0.001**
CDT	4.8 (0.4)	3.7 (1.6)ł	4.7 (0.6)	3.8 (1.2)ǂ	0.4	0.6	7.7	**<0.001**

ł and ǂ Represent the significant difference between YHC and OHC, as well as EOAD and LOAD ($p<0.05$), respectively. Interactive effects comprise the factors of onset age (<65 or ≥65 years) and disease status (controls or patients). Abbreviations: YHC, young healthy controls; EOAD, early-onset Alzheimer's disease; OHC, old healthy controls; LOAD, late-onset Alzheimer's disease; CDR global/sum, Clinical Dementia Rating, global score and sum score of box; MMSE, Mini-Mental State Examination; GDS, Geriatric Depression Scale; LM, Logical Memory; TMT-A/B, Trail Making Test, part A/B; SVF, Semantic Verbal Fluency.

NINCDS/ADRDA (National Institute of Neurological and Communicative Disorders and Stroke and the Alzheimer's Disease and Related Disorders Association) criteria [5]. Additionally, the neurologist also evaluated the neurological history, blood biochemical examination, and neuropsychological scales to exclude dementia from other causes. The age of disease onset was identified by the interview conducted with the patient's family members or caregivers. Regarding the ADNI database, neurologists from multiple cooperation institutes made the AD diagnosis. We downloaded the "diagnosis summary" from LONI (https://ida.loni.usc.edu) in 2018 July. Consistent with previous studies, we dichotomized AD patients into early- and late-onset groups (age at onset <65 or ≥ 65 years, respectively) [6, 7].

In both databases, we defined YHC and OHC as having a CDR of 0, an MMSE between 24 and 30 (inclusive), a WMS-LM, delayed recall (≥ 9 for subjects having ≥ 16 years education; ≥ 5 for subjects having 8-15 years education; and ≥ 3 for subjects having ≤ 7 years education); absence of clinical depression (GDS < 6) and dementia symptom. Further, we excluded subjects with the following manifestations: significant neurological, psychiatric, and medical illness; severe head trauma history; application of non-AD-related medication potentially influence cerebral function; clinical depression; drug or alcohol abuse.

Supplementary Materials 2

Repeated FBA based on the matched sample size of both databases

Due to the differences in the sample sizes of two independent databases, different statistical effects may contribute to the repeated result difference between databases. To eliminate the potential factor, we compressed the sample of the ZJU database to the same size as the ADNI database. There is still no significant differences in age, gender, education, general cognitive, and disease severity between groups of patients and controls in the compressed ZJU database. Then, we re-performed a whole brain-based FBA in the ZJU database after sample reduction (FWE-corrected, $p < 0.05$, 5000 permutations) [8].

Although the affected regions got smaller, the trend of results remained unchanged. We found that EOAD had widespread FD decreases in the splenium of corpus callosum (SCC), left fornix-HP, and bilateral dorsal and ventral cingulum relative to YHC. Additionally, EOAD had an FC decrease in the bilateral dorsal cingulum relative to YHC. Regarding the FDC, we found that EOAD patients had a widespread decrease in the bilateral dorsal and ventral cingulum, and left fornix-HP relative to YHC. By contrast, we found that LOAD patients had a marked FD decrease in the bilateral ventral cingulum and FC decrease in bilateral dorsal and ventral cingulum relative to OHC. No difference in FDC existed between LOAD and ONC.

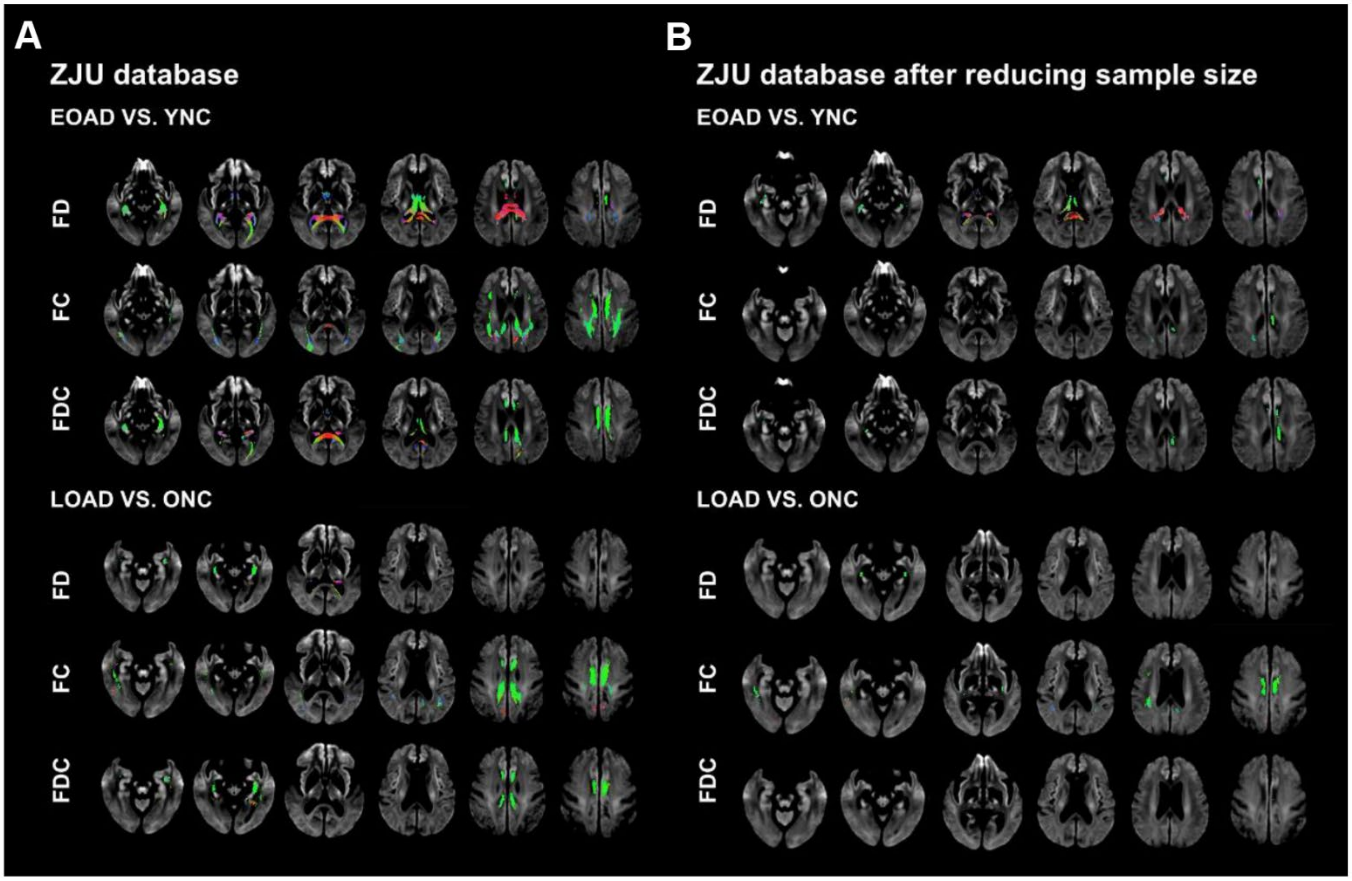

Supplementary Figure 1. The fiber tract-specific reduction in EOAD/LOAD versus controls from whole-brain FBA. (**A**, **B**) Represent results from the database of ZJU and ZJU after sample reduction, respectively. We color-coded the significant streamlines (patients versus controls) by streamline orientation (left-right: red; inferior-superior: blue; anterior-posterior: green). Abbreviation: FBA, fixel-based analysis; FD, fiber density; FC, fiber bundle cross-section; FDC, fiber density and bundle cross-section.

Supplementary Table 2. Demographics of ZJU database after sample reduction.

Group	YHC	EOAD	OHC	LOAD	Interaction		ANOVA	
	n=31	n=17	n=34	n=30	F/χ^2	p	F/χ^2	p
Age	62.1 (1.6)	61.2 (2.3)	74.9 (3.7)	76.1 (3.0)	3.5	0.06	206	**<0.001**
Education	11.0 (4.0)	10.6 (3.0)	10.6 (4.2)	10.8 (4.0)	0.1	0.7	0.1	0.9
Sex, F/M	18/13	10/7	15/19	16/14	0.2	0.7	1.6	0.7
GDS	1.6 (1.9)	1.7 (1.8)	1.1 (1.6)	1.3 (1.1)	0.01	0.9	0.8	0.5
CDR global	0 (0)	1.0 (0.3)	0 (0)	1.0 (0.5)	0.1	0.8	138.7	**<0.001**
CDR sum	0 (0)	4.7 (2.4)	0 (0.1)	4.5 (3.5)	0.1	0.8	47.0	**<0.001**
MMSE	28.3 (1.6)	21.1 (3.1)	28.4 (1.6)	20.2 (3.6)	0.9	0.3	89.0	**<0.001**
LM delay	8.1 (4.2)	0.6 (1.5)	8.2 (3.7)	0.3 (0.8)	0.01	0.9	56.3	**<0.001**
TMT-A	69.0 (27.4)	86.6 (39.1)	69.8 (24.4)	105.7 (36.5)	0.9	0.4	9.4	**<0.001**
TMT-B	163.1 (74.4)	228.8 (79.4)	188.0 (62.6)	263.7 (70.3)	0.2	0.6	11.8	**<0.001**
SVF	17.0 (3.8)	12.4 (5.4)	16.7 (4.0)	8.6 (5.2)	2.9	0.1	23.4	**<0.001**
CDT	4.1 (0.7)	3.1 (0.6)	4.2 (0.5)	3.4 (0.6)	0.7	0.4	17.5	**<0.001**

ǂ and ǂ Represent the significant difference between YHC and OHC, as well as EOAD and LOAD (p<0.05), respectively. Interactive effects comprise the factors of onset age (<65 or ≥65 years) and disease status (controls or patients). Abbreviations: YHC, young healthy controls; EOAD, early-onset Alzheimer's disease; OHC, old healthy controls; LOAD, late-onset Alzheimer's disease; CDR, Clinical Dementia Rating; MMSE, Mini-Mental State Examination; GDS, Geriatric Depression Scale; LM, Logical Memory; TMT-A/B, Trail Making Test, part A/B; SVF, Semantic Verbal Fluency.

Supplementary Materials 3

Effects of white matter hyperintensities on fixel-based analysis

Increasing evidence shows that AD is a multifactorial and heterogeneous disease with multiple contributors to its pathophysiology, including cerebrovascular disease [9]. Among them, WMH is the typical imaging marker of cerebral small vascular disease (CSVD) [10]. We thus calculated WMH through semi-quantitative visual assessment [11]. We found that the elderly group (LOAD and LONC) had more WMH burden than the young group (EONC and EOAD); while dementia group (EOAD and LOAD) had more WMH burden than the healthy group (EONC and LONC).

Supplementary Table 3. The distribution of WMH burden among four groups in two databases.

ZJU database (n, %)	WMH Fazekas score (0, 1, 2, 3)
EOAD	9 (29.0), 17 (54.8), 3 (9.7), 2 (6.5)
EONC	26 (40.6), 35 (54.7), 2 (3.13), 1 (1.6)
LOAD	2 (4.4), 17 (37.8), 10 (22.2), 16 (35.6)
LONC	11 (23.9), 17 (37.0), 7 (15.2), 11 (23.9)
ADNI database (n, %)	WMH Fazekas score (0, 1, 2, 3)
EOAD	12 (70.6), 4 (23.5), 1 (5.8), 0 (0)
EONC	14 (45.2), 13 (41.9), 2 (6.5), 2 (6.5)
LOAD	10 (33.3), 4 (13.3), 11 (36.7), 5 (16.7)
LONC	17 (50.0), 10 (29.4), 6 (17.6), 1 (2.9)

Considering that many difference regions in FBA results partially overlapped with paraventricular WMH, we further re-performed FBA analysis with WMH as a covariable. After adjustment for WMH, our results show that the trend in FBA outcomes remained mostly unchanged in both the databases of ZJU and ADNI, but the range of differences between groups became smaller. Basically, consistent with recent findings, our results suggest that WMH does contribute to the microstructural lesions in AD patients to some extent [12]. Thus, it is necessary to take CSVD into account in future AD studies.

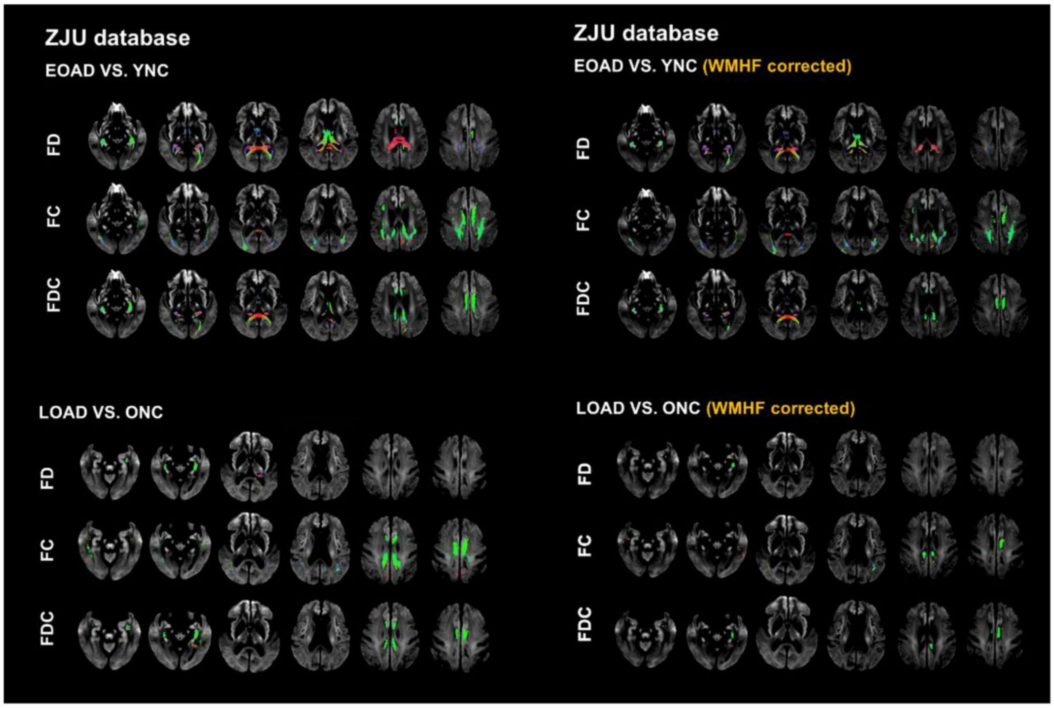

Supplementary Figure 2. The location reference and fiber tract-specific reduction in EOAD/LOAD versus controls (ZJU database) from whole-brain fixel-based analysis, results corrected by Fazekas WMH score. We color-coded the significant streamlines by the effect size expressed as a percentage relative to the control groups. Abbreviations: ZJU, Zhejiang University; FD, fiber density; FC, fiber bundle cross-section; FDC, fiber density and bundle cross-section.

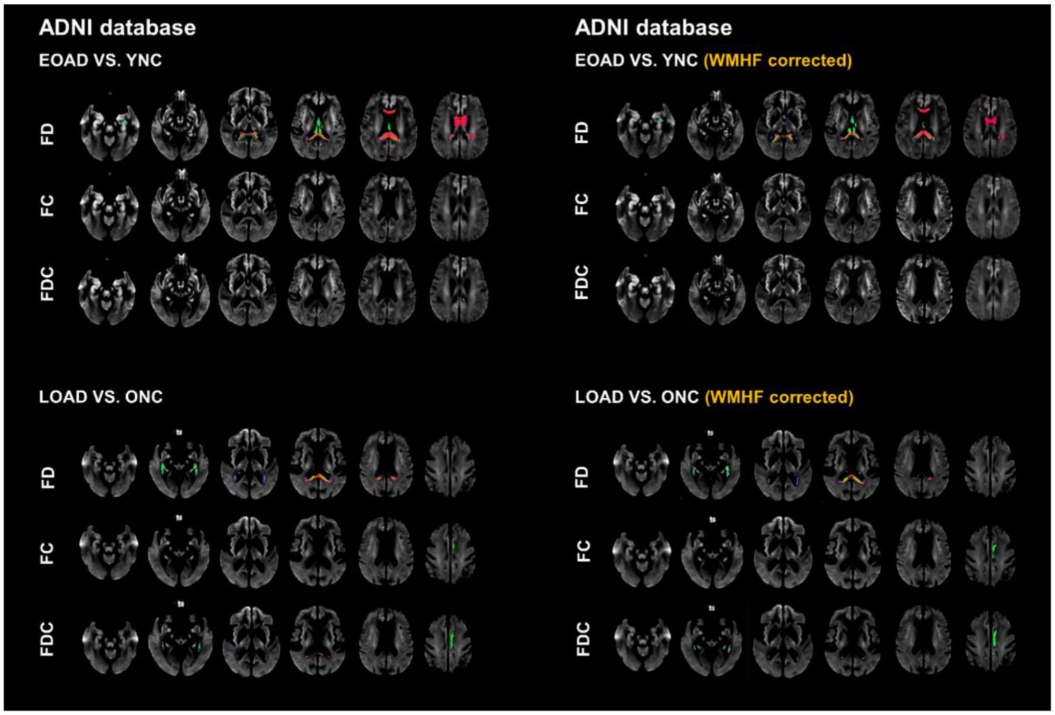

Supplementary Figure 3. The location reference and fiber tract-specific reduction in EOAD/LOAD versus controls (ADNI database) from whole-brain fixel-based analysis, results corrected by Fazekas WMH score. We color-coded the significant streamlines by the effect size expressed as a percentage relative to the control groups. Abbreviations: ADNI, Alzheimer's Disease Neuroimaging Initiative; FD, fiber density; FC, fiber bundle cross-section; FDC, fiber density and bundle cross-section.

Supplementary Materials 4

Association between fixel-based analysis metrics and cognitive/PET data across groups

Across four groups (EOAD, YHC, LOAD, and OHC), we correlated both the mean FD and FC of significant tracts in group differences analyses with the cognitive score (uncorrected, $p < 0.001$, controlling age and gender). Additionally, in the ADNI database, we also correlated both the mean FD and FC of significant tracts in group differences analyses with the PET data (uncorrected, $p < 0.001$, controlling age and gender).

ZJU database

Regarding the WM microstructural metric, we found that MMSE was related with FD in left PTR (r = 0.23); CDT was related with FD in the bilateral PTR (r = 0.26 and 0.24, respectively); delay recall was related with FD in the left ventral cingulum (r = 0.27); SVF was related with FD in the left ventral cingulum (r = 0.26) and left ILF/IFOF (r = 0.25); TMT-A was related with FD in the SCC (r = -0.24), bilateral dorsal cingulum (r = -0.24/-0.23, respectively), and left ventral cingulum (r = -0.27); TMT-B was related with FD in the ventral cingulum (r = -0.23). Regarding the macrostructural metric, we found that MMSE was related with FC in the dorsal cingulum (r = 0.24), delay recall was related with FC in the fornix column and body (r = -0.29), bilateral fornix HP (r = -0.25/-0.25, respectively); while TMT-B was related with FC in the right PTR (r = -0.23).

Within EOAD patients, we found that TMT-A was related with the left dorsal cingulum (r = -0.59); TMT-B was related with SCC (r = -0.57) and left dorsal cingulum (r = -0.57). Bycontrast, within LOAD patients, we found that SVF was related to left ventral cingulum (r = 0.41).

ADNI database

Regarding the WM microstructural metric, we found that MMSE was related with FD in the SCC (r = 0.38), fornix column and body (r = 0.40), left PTR (r = 0.35); CDR was related with FD in the SCC (r = -0.31), fornix column and body (r = -0.36), right dorsal cingulum (r = -0.31), left ILF/IFOL (r = -0.35); SVF was related with FD in the SCC (r = 0.37), fornix column and body (r = 0.46), and bilateral PRT (r = 0.29/0.49, respectively); TMT-B was related with FD in the SCC (r = -0.31) and fornix column and body (r = -0.41), left PRT (r = -0.32). Regarding the WM macrostructural metric, we found that MMSE was related with FC in the fornix column and body (r = -0.37), SVF was related with FC in the left dorsal cingulum (r = 0.35), TMT-A was related with FC in the SCC (r = -0.36), bilateral dorsal cingulum (r = -0.35/-0.48, respectively), and left PTR (r = -0.33); TMT-B was related with FC in the SCC (r = -0.37). Further, we found mean tau retention of Braak I-II was related with FC in the right PTR (r = 0.83).

Within EOAD patients, we found that IST was related with the fornix column and body (r=0.71). Bycontrast, within LOAD patients, we found that SVF was related with the left PTR (r=0.55), SCC (r=0.49), right dorsal cingulum (r=0.52), and left dorsal cingulum (r=0.62); TMT B was related with SCC (r=-0.49), left (r=-0.54) and right (r=-0.56) PTR.

Supplementary Materials 5

Braak ROIs defined by Freesurfer [13]

Braak I-II: bilateral entorhinal and hippocampus

Braak III-IV: bilateral parahippocampal gyrus, fusiform, lingual, amygdala, middle temporal, thalamus, caudantcing, rostantcing, postcing, isthmuscing, insula, inferior temporal, temporal pole.

Braak V-VI: bilateral superior frontal, lateral orbitofrontal, medial orbitofrontal, frontal pole, caudal middle frontal, rostral middle frontal, pars opercularis, pars orbitalis, pars triangularis, lateral occipital, parietal supramarginal, parietal inferior, superior temporal, parietal superior, precuneus, bank superior temporal sulcus, accumbens, tranvtemp, pericalcarine, postcentral, cuneus, precentral, paracentral.

More detailed information are available in https://adni.bitbucket.io/reference/docs/UCBERKELEYAV1451/UCBERKELEYAV1451_Methods_20171114.pdf

Supplementary References

1. Li KC, Luo X, Zeng QZ, Xu XJ, Huang PY, Shen ZJ, Xu JJ, Zhou J, Zhang MM. Distinct patterns of interhemispheric connectivity in patients with early- and late-onset Alzheimer's disease. Front Aging Neurosci. 2018; 10:261. https://doi.org/10.3389/fnagi.2018.00261 PMID:30237764

2. Folstein MF, Robins LN, Helzer JE. The mini-mental state examination. Arch Gen Psychiatry. 1983; 40:812.

https://doi.org/10.1001/archpsyc.1983.01790060110016
PMID:6860082

3. Morris JC. The clinical dementia rating (CDR): current version and scoring rules. Neurology. 1993; 43:2412–14.
https://doi.org/10.1212/wnl.43.11.2412-a
PMID:8232972

4. Sheikh JI, Yesavage JA. Geriatric Depression Scale (GDS): recent evidence and development of a shorter version. Clinical Gerontologist: The Journal of Aging and Mental Health. 1986.
https://doi.org/10.1300/J018v05n01_09

5. Dubois B, Feldman HH, Jacova C, Dekosky ST, Barberger-Gateau P, Cummings J, Delacourte A, Galasko D, Gauthier S, Jicha G, Meguro K, O'brien J, Pasquier F, et al. Research criteria for the diagnosis of Alzheimer's disease: revising the NINCDS-ADRDA criteria. Lancet Neurol. 2007; 6:734–46.
https://doi.org/10.1016/S1474-4422(07)70178-3
PMID:17616482

6. Canu E, Agosta F, Spinelli EG, Magnani G, Marcone A, Scola E, Falautano M, Comi G, Falini A, Filippi M. White matter microstructural damage in Alzheimer's disease at different ages of onset. Neurobiol Aging. 2013; 34:2331–40.
https://doi.org/10.1016/j.neurobiolaging.2013.03.026
PMID:23623599

7. Cho H, Choi JY, Lee SH, Lee JH, Choi YC, Ryu YH, Lee MS, Lyoo CH. Excessive tau accumulation in the parieto-occipital cortex characterizes early-onset Alzheimer's disease. Neurobiol Aging. 2017; 53: 103–11.
https://doi.org/10.1016/j.neurobiolaging.2017.01.024
PMID:28254589

8. Nichols TE, Holmes AP. Nonparametric permutation tests for functional neuroimaging: a primer with examples. Hum Brain Mapp. 2002; 15:1–25.
https://doi.org/10.1002/hbm.1058 PMID:11747097

9. Sweeney MD, Montagne A, Sagare AP, Nation DA, Schneider LS, Chui HC, Harrington MG, Pa J, Law M, Wang DJJ, Jacobs RE, Doubal FN, Ramirez J, et al. Vascular dysfunction-The disregarded partner of Alzheimer's disease. Alzheimers Dement. 2019; 15:158–167.
https://doi.org/10.1016/j.jalz.2018.07.222
PMID:30642436

10. Wardlaw JM, Smith C, Dichgans M. Small vessel disease: mechanisms and clinical implications. Lancet Neurol. 2019; 18:684–96.
https://doi.org/10.1016/S1474-4422(19)30079-1
PMID:31097385

11. Fazekas F, Chawluk JB, Alavi A, Hurtig HI, Zimmerman RA. MR signal abnormalities at 1.5 T in Alzheimer's dementia and normal aging. AJR Am J Roentgenol. 1987; 149:351–6.
https://doi.org/10.2214/ajr.149.2.351 PMID:3496763

12. Finsterwalder S, Vlegels N, Gesierich B, Araque Caballero MÁ, Weaver NA, Franzmeier N, Georgakis MK, Konieczny MJ, Koek HL, and Dominantly Inherited Alzheimer Network (DIAN), Karch CM, Graff-Radford NR, Salloway S, Oh H, et al. Small vessel disease more than Alzheimer's disease determines diffusion MRI alterations in memory clinic patients. Alzheimers Dement. 2020; 16:1504–14.
https://doi.org/10.1002/alz.12150 PMID:32808747

13. Fischl B. FreeSurfer. Neuroimage. 2012; 62:774–81.
https://doi.org/10.1016/j.neuroimage.2012.01.021
PMID:22248573
